# Self-renewal and chemotherapy resistance of p75^NTR ^positive cells in esophageal squamous cell carcinomas

**DOI:** 10.1186/1471-2407-9-9

**Published:** 2009-01-10

**Authors:** Sheng-Dong Huang, Yang Yuan, Xiao-Hong Liu, De-Jun Gong, Chen-Guang Bai, Feng Wang, Jun-Hui Luo, Zhi-Yun Xu

**Affiliations:** 1 Institute of Cardiothoracic Surgery, Changhai Hospital, Second Military Medical University, Shanghai, PR China; 2 Department of Pathology, Changhai Hospital, Second Military Medical University, Shanghai, PR China

## Abstract

**Background:**

p75^NTR ^has been used to isolate esophageal and corneal epithelial stem cells. In the present study, we investigated the expression of p75^NTR ^in esophageal squamous cell carcinoma (ESCC) and explored the biological properties of p75^NTR+ ^cells.

**Methods:**

p75^NTR ^expression in ESCC was assessed by immunohistochemistry. p75^NTR+ ^and p75^NTR- ^cells of 4 ESCC cell lines were separated by fluorescence-activated cell sorting. Differentially expressed genes between p75^NTR+ ^and p75^NTR- ^cells were determined by real-time quantitative reverse transcription-PCR. Sphere formation assay, DDP sensitivity assay, ^64^copper accumulation assay and tumorigenicity analysis were performed to determine the capacity of self-renewal, chemotherapy resistance and tumorigenicity of p75^NTR+ ^cells.

**Results:**

In ESCC specimens, p75^NTR ^was found mainly confined to immature cells and absent in cells undergoing terminal differentiation. The percentage of p75^NTR+ ^cells was 1.6%–3.7% in Eca109 and 3 newly established ESCC cell lines. The expression of Bmi-1, which is associated with self-renewal of stem cells, was significantly higher in p75^NTR+ ^cells. p63, a marker identified in keratinocyte stem cells, was confined mainly to p75^NTR+ ^cells. The expression of CTR1, which is associated with cisplatin (DDP)-resistance, was significantly decreased in p75^NTR+ ^cells. Expression levels of differentiation markers, such as involucrin, cytokeratin 13, β1-integrin and β4-integrin, were lower in p75^NTR+ ^cells. In addition, p75^NTR+ ^cells generated both p75^NTR+ ^and p75^NTR- ^cells, and formed nonadherent spherical clusters in serum-free medium supplemented with growth factors. Furthermore, p75^NTR+ ^cells were found to be more resistant to DDP and exhibited lower ^64^copper accumulation than p75^NTR- ^cells.

**Conclusion:**

Our results demonstrated that p75^NTR+ ^cells possess some characteristics of CSCs, namely, self-renewal and chemotherapy resistance. Chemotherapy resistance of p75^NTR+ ^cells may probably be attributable to decreased expression of CTR1.

## Background

The "cancer stem cell" theory has aroused increasing interest in the field of oncology [1-4]. According to this theory, cancer stem cells (CSCs), which account for a very small proportion of tumor tissue, have the self-renewal property typical of normal stem cells. CSCs rarely divide, but they are able to produce fast-proliferating daughter cells. Previous studies have demonstrated the existence of CSCs in a variety of tumors including liver carcinoma [5,6], brain tumors [1,2], breast carcinoma [7], lung carcinoma [8], colorectal carcinoma [9], pancreatic carcinoma [10], and thyroid tumors [11]. In addition to the basic stem cell properties, CSCs also display high resistance to radiation and conventional chemotherapy. Bao et al [12] reported that CD133 positive CSCs contributed to glioma radioresistance through preferential activation of DNA damage checkpoint response and increased capacity of DNA repair. Liu et al [13] also reported that CD133 positive CSCs in brain glioblastoma showed strong chemoresistance attributable to higher expression of ABCG2 and MGMT. These studies strongly support the cancer stem cell theory that CSCs are the underlying cause of recurrence and metastasis of tumors.

Low-affinity neurotrophin receptor (p75^NTR^), a member of tumor necrosis factor superfamily [14,15], has been shown to paradoxically mediate neuronal survival and differentiation or apoptotic cell death, depending on the environment of the cells [16]. Okumura et al [17] reported that p75^NTR+ ^esophageal epithelial cells were stem cells due to their capacity of proliferation, self-renewal and multidirectional differentiation. p75^NTR ^was also used for screening mouse testis peritubular smooth muscle precursors [18], rat fat multipotential stem cells [19], and human corneal epithelial progenitor cells [20]. In the present study, we found that p75^NTR+ ^esophageal squamous cell carcinomas (ESCC) cells exhibited properties of CSCs in terms of self-renewal and chemotherapy resistance.

## Methods

### ESCC specimens and immunohistochemistry

A total of 100 patients with histopathologically confirmed ESCC who underwent surgery at Changhai Hospital between 2005 and 2006 were selected. Informed consent was obtained from the patients or their guardians. No preoperative history of radio-therapy or chemotherapy was reported in any of the patients.

Antibodies used in this study included: mouse anti human p75^NTR ^(Upstate Corporation, USA), involucrin (Santa cruz Corporation, USA), p63 (DAKO Corporation, Denmark) and ki-67 (DAKO Corporation, Denmark). For antigen retrieval, the slides were treated with boiling 10 mM citrate buffer (pH 6.0) for 25 min. Immunohistochemical staining was performed using EnVision™ Kit (HRP, DAKO Corporation, Denmark). All slides were evaluated independently by two investigators (XHL. and CGB.) without prior knowledge of the clinical information of the patients. The samples were divided into two groups according to the percentage of p75^NTR ^staining-positive cells, tumors were classified as positive if >10% tumor cells were stained and negative if ≤ 10% tumor cells were stained[21]. The ki-67 index was defined as the percentage of ki-67 positive tumor cell nuclei.

### Cell source and culture condition

Esophageal carcinoma cell line Eca109 was purchased from Shanghai Cell Biology Institute of the Chinese Academy of Sciences. Eca109-eGFP was established by lentiviral vector (Invitrogen Corporation, USA). Cells were cultured in DMEM containing 10% fetal bovine serum at 37°C, 5% CO_2_. Three cell lines (SHEC-1, SHEC-4 and SHEC-5) were newly established. In brief, surgically removed fresh specimens were rinsed with serum-free RPMI-1640 (Invitrogen Corporation, USA) for 3 times, cut into 1 mm^3 ^tissue masses and incubated in enzymatic dissociation in DMEM containing 1 mg/mL collagenase IV (Invitrogen Corporation, USA) for 4–6 h at 37°C. The isolated cells were cultured in DMEM containing 10% fetal bovine serum in a humidified 5% CO_2 _atmosphere at 37°C. Three cell lines were established by serial passage and designated SHEC-1, SHEC-4 and SHEC-5, respectively.

### Flow cytometry and fluorescence-activated cell sorting

Tumor cells were harvested and adjusted to a concentration of 1 × 10^6 ^cells/ml with Buffer1 (phosphate buffered saline containing 0.5% bovine serum albumin and 2 mM EDTA). The cells were incubated with primary antibody for 2 h at 4°C. After washing with Buffer1 twice, the cells were resuspended in 500 μl Buffer1, to which PE-conjugated goat anti mouse IgG (BD PharMingen Corporation, USA) was added. The samples were then incubated away from light for 15 min at 4°C. Following staining, the samples were analyzed using a FACSCalibur flow cytometer and CellQuest software (BD Biosciences, San Jose, CA). Primary antibodies were: mouse anti human p75^NTR^, involucrin, β1-integrin (Santa cruz Corporation, USA) and cytokeratin 13 (Santa cruz Corporation, USA). Fluorescence-activated cell sorting (FACS) of p75^NTR+^and p75^NTR- ^cells was performed on a Cytomation MoFlo cytometer (DakoCytomation Corporation, Denmark). The top 25% most brightly stained cells were isolated as p75^NTR+ ^cells. Cells incubated with PE-conjugated antibodies were used as controls.

### RNA isolation and cDNA synthesis

Total RNA of tumor cells was extracted by RNA4PCR kit (Amibion Corporation, USA) according to the manufacturer's instructions. RNA was dissolved in 50 μl distilled water containing 0.1% diethylpyrocarbonate and quantitated at OD_260 _by spectrophotometry.

### Real-time quantitative reverse transcription-PCR

Total RNA was used as the template to amplify Bmi-1, p63, involucrin, cytokeratin 13, β1-integrin and β4-integrin and CTR1 by real-time quantitative reverse transcription-PCR (real-time qPCR). G6PDH was used as internal control. The following primer sequences were used, and expected product sizes were noted in parentheses.

Bmi-1: 5'-ggagaccagcaagtattgtccttttg-3' and 5'-cattgctgctgggcatcgtaag-3' (348 bp);

p63: 5'-cagacttgccaaatcatcc-3' and 5'-cagcattgtcagtttcttagc-3' (120 bp);

involucrin: 5'-tcctcctccagtcaataccc-3' and 5'-gctgatccctttgtgtt-3' (283 bp);

cytokeratin 13: 5'-ccatgaagaggtgagcggggattg-3' and 5'-ctgtggggatgggaaaggaagatgtg-3' (200 bp);

β1-integrin: 5'-gtggttgctggaattgttctta-3' and 5'-agtgttgtgggatttgcac-3' (219 bp);

β4-integrin: 5'-atagagtcccaggatggagga-3' and 5'-gtggtggagatgctgctgta-3' (97 bp);

CTR1: 5'-aggactcaagatagcccgagaga-3' and 5'-tggtcctgggacaggcatgg-3' (78 bp).

G6PDH: 5'-atcgaccactacctgggcaa-3' and 5'-ttctgcatcacgtcccgga-3' (191)

### Sphere formation assay

Tumor cells were resuspended to 1 × 10^4 ^cells/ml in serum-free medium (keratinocyte-SFM medium, with addition of penicillin 100 IU/ml, streptomycin 100 μg/ml, epidermal growth factor 5 ng/ml, bovine pituitary extract 70 μg/ml, hydrocortisonum 0.5 μg/ml and regular insulin 5 μg/ml in sequence). The cultures were monitored for the sphere formation within 3 weeks. To passage the spheres, medium was aspirated off and spheres were harvested with the TransferMan NK 2 micromanipulator (Eppendorf). After trypsin digestion, single cell suspension was prepared by mechanical dissociation. To determine the differentiation of p75^NTR+ ^cells, single viable floating p75^NTR+ ^cells were collected and resuspended to 1 × 10^3 ^cells/mL in keratinocyte-SFM medium containing 10% fetal bovine serum. The expression of involucrin and cytokeratin 13 was detected by immunocytochemistry.

### DDP sensitivity assay

Stock solution of 1 mg/ml DDP (Sigma-Aldrich, USA) was prepared in dimethylformamide and stored at -20°C for no longer than 3 days. Isolated p75^NTR+ ^and p75^NTR- ^cells were seeded into 96-well culture plates (5 × 10^2 ^cells/well) respectively, and incubated in an atmosphere containing 5% CO_2 _for 24 h at 37°C. In our pilot study, DDP inhibited the viability of Eca109 cells in a concentration-dependent manner with an apparent IC10 value of 0.23 μg/ml, IC50 value of 0.47 μg/ml and IC90 value of 0.68 μg/ml. In the present study, the cells were treated with DDP at concentration of 0.5 and 1 μg/ml based on the pilot cytotoxicity study. MTT assay was performed to determine the viability of the cells after they were exposed to DDP for 4 days.

Furthermore, cell mixture composed of 10% p75^NTR+ ^Eca109-eGFP and 90% p75^NTR- ^Eca109 cells were cultured for 2 days and then exposed to 0.5 and 1 μg/ml DDP for 4 days respectively. The percentage of Eca109-eGFP cells was analyzed by flow cytometry.

### ^64^copper accumulation assay

Isolated p75^NTR+ ^and p75^NTR- ^cells were seeded into 6-well culture plates (1 × 10^5 ^cells/well), and incubated in an atmosphere containing 5% CO_2 _for 24 h at 37°C. The medium was replaced with 1 ml fresh medium containing 2 μM ^64^CuSO_4_, and the cells were incubated in 5% CO_2 _for 0.5, 1.0, and 1.5 h at 37°C. After that, the plates were placed on ice and rinsed 3 times with 6 ml ice-cold PBS. Then 500 μl cell lysis buffer (0.1% Triton X-100 and 1% SDS in PBS) was added to the wells and the lysate was harvested by scraping the dish twice. The samples were transferred to tubes for γ counting on a Gamma 5500 B counter (Beckman Coulter, Inc., Fullerton, CA). Samples unexposed to ^64^CuSO_4 _were measured as control.

### Xenograft tumorigenicity assay

Six to eight-week-old female congenitally immune-deficient nonobese diabetic/severe combined immune-deficiency (NOD/SCID) mice were randomly divided into different groups and maintained under standard conditions according to the institution's guidelines. Various numbers of p75^NTR+ ^and p75^NTR- ^Eca109 cells were suspended in 200 μl serum-free DMEM or in 200 μl serum-free DMEM/Matrigel (1:1), and injected subcutaneously into NOD/SCID mice. The mice were killed 10–16 weeks after tumor cell inoculation. Animals with no signs of tumor burden were further examined by autopsy to confirm that there was no tumor development.

### Statistical analysis

For comparisons of gender, necrosis, distant metastasis and paraesophageal lymph node metastasis between p75^NTR ^positive and negative groups, chi-square test was performed. The correlation between p75^NTR ^expression and pathological grade was analyzed by Cochran-Mantel-Haenszel Statistics. Age of the patients and tumor diameter between two groups was compared by independent Student's t test. Other data obtained from experiments were expressed as mean ± SD ($\bar{\text{x}}$ ± s) and tested by Student's t test. Statistical analyses were conducted with SAS Statistical Software (version 9.1.3). *P *< 0.05 was considered significant.

## Results

### Expression of p75^NTR ^in ESCC specimens

**Figure 1:**
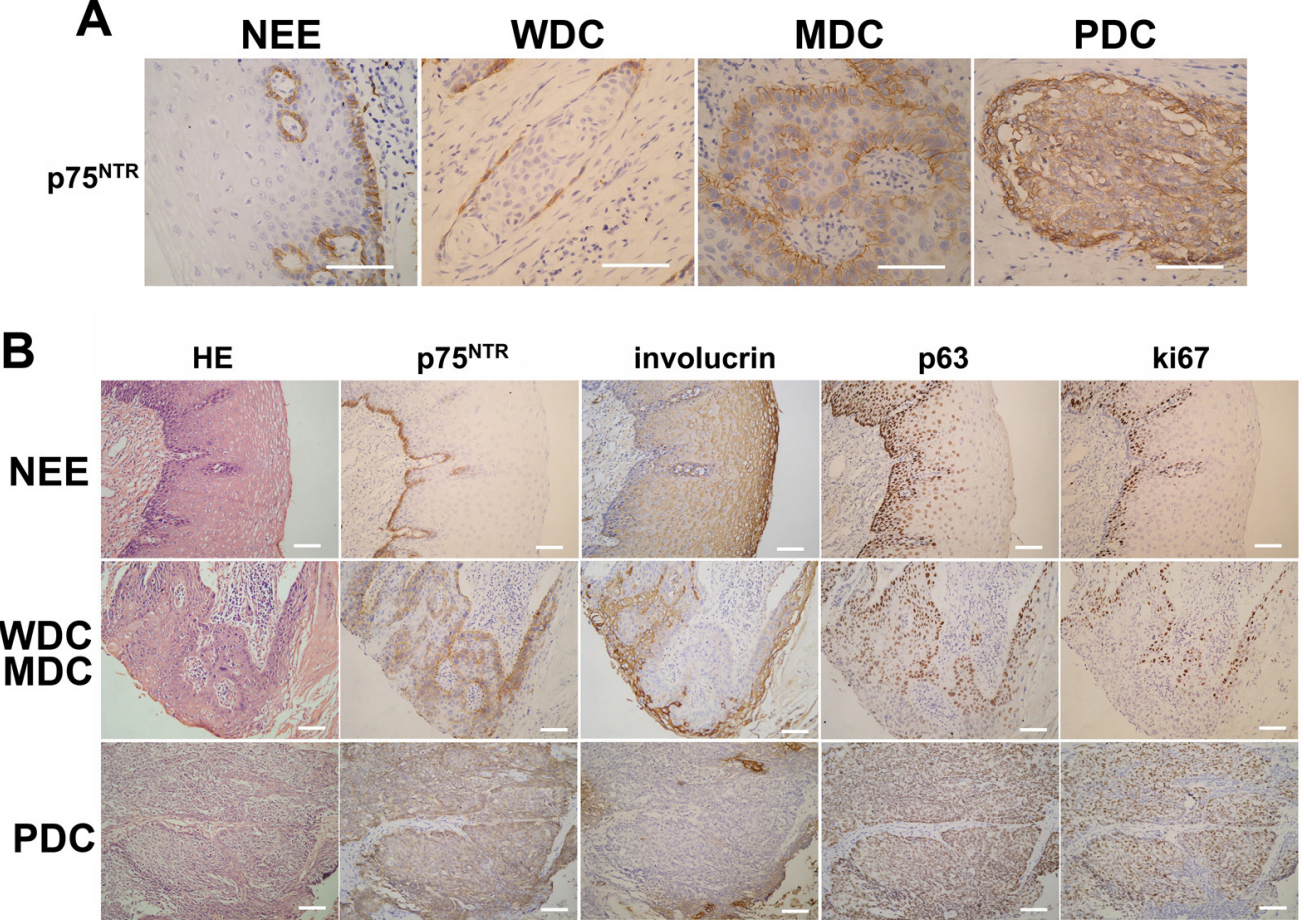
**Parallel sections from normal esophageal epithelial and ESCC specimen**. (A) In NEE, p75^NTR ^was located mainly in the basal layer; in WDC, p75^NTR ^positive staining was apparent in the first one to two layers from the infiltrative margin, areas exhibiting stratified squamous pearl formation were negative for p75^NTR^; in MDC, p75^NTR ^was expressed in wider range from the margin of the tumors; p75^NTR ^was diffusely distributed in PDC.(B) In NEE, basal cells coexpressed p75^NTR ^and p63, and very few p75^NTR+ ^cells expressed ki-67; in WDC and MDC, p75^NTR+ ^cells were at some distance surrounding involucrin^+^cells where most of the cells were coexpressed p63, and a small proportion of p75^NTR+ ^cells expressed ki-67; in PDC, few cells were positive for involucrin, more cells contained both p63 and p75^NTR^, and their distribution was chaotic, a large number of p75^NTR+ ^cells stained brightly for ki-67. (Bars = 200 μm)

As shown in Figure 1A, p75^NTR ^was found on the membrane of the basal cells in normal esophageal epithelium (NEE) and absent in the spinous layer. In well differentiated cases (WDC) and moderately differentiated cases (MDC), p75^NTR ^staining was apparent in the first few layers adjacent to the infiltrative margin of the tumors. In contrast, in poorly differentiated cases (PDC), p75^NTR ^was diffusely distributed.

In contrast to p75^NTR^, involucrin staining was located in the spinous layer of NEE. Parallel sections from WDC and MDC showed that p75^NTR+ ^cells were located at some distance from the terminally differentiated cells stained brightly for involucrin, and generally absent in the zones immediately surrounding the involucrin^+ ^cells. In PDC, there were few cells positive for involucrin, most of the cells were stained very brightly for p75^NTR^. Distribution of the p75^NTR+ ^cells was irregular. In NEE, only a small proportion of p75^NTR+ ^cells expressed ki-67. In ESCC specimens, however, the proportion of p75^NTR ^and ki-67 coexpressing cells increased. In addition, p75^NTR ^and p63 had similar tissue distribution both in NEE and in ESCC specimens (Figure 1B).

The correlation between p75^NTR ^expression and various prognostic factors were investigated (Table 1). p75^NTR ^expression correlated with age (P = 0.008), tumor diameter (P = 0.004) and pathological grade (P = 0.001). There was no significant correlation between p75^NTR ^expression and other factors such as gender, ki-67 index, necrosis, distant metastasis and paraesophageal lymph node metastasis.

**Table 1:** Relationship between p75^NTR ^expression and clinicopathologic characteristics

clinical factors	squamous cell carcinomas			
	
	p75^NTR^-positive	p75^NTR^-negative	*test*	*P*
**Total**	62	38		
**age (year)**	62.95 ± 9.27	58.13 ± 7.524	2.71	0.008
**gender**				
Male	47/62 (75.8%)	9/38 (23.7%)	0.32	0.569
Female	15/62 (24.2%)	29/38 (76.3%)		
**tumor diameter (cm)**	5.38 ± 1.76	4.16 ± 1.85	3.30	0.001
**KI**				
high	40/62 (64.5%)	24/38 (63.2%)	0.02	0.891
Low	22/62 (35.5%)	14/38 (36.8%)		
**necrosis**	17/62 (27.4%)	14/38 (36.8%)	0.98	0.323
*distant metastasis	16/62 (25.8%)	10/38 (26.3%)	0.00	0.955
**Para-esophageal lymph node metastasis**	29/62 (46.8%)	19/38 (50.0%)	0.10	0.754
**pathological grade**				
poorly differentiated	6 (9.7%)	10 (26.3%)	11.78	0.001
moderately differentiated	26 (41.9%)	22 (57.9%)		
well differentiated	30 (48.4%)	6 (15.8%)		

* Distant metastasis involved supraclavicular lymph node, abdominal lymph node, liver, lung, trachea and subcutaneous of the back.

### Growth characteristics of p75^NTR+ ^cells

**Figure 2:**
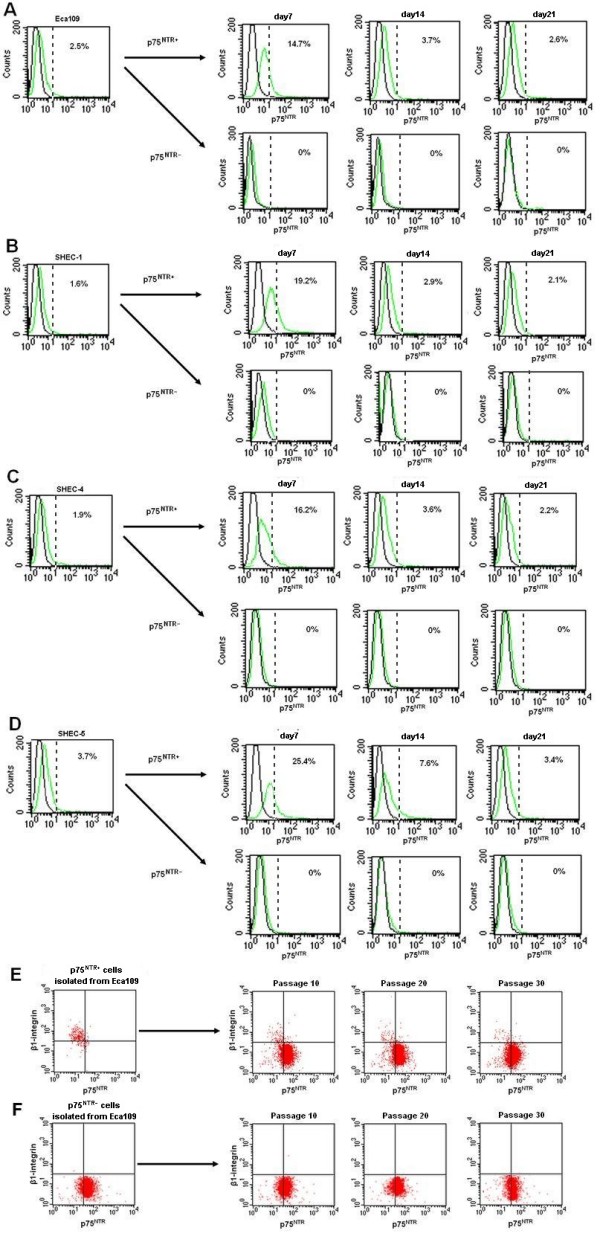
**p75^NTR ^expression in ESCC cell line**. p75^NTR+ ^cells made up 2.5% in Eca109 (A), 1.6% in SHEC-1 (B), 1.9% in SHEC-4 (C) and 3.7% (D) in SHEC-5. Cells derived from p75^NTR+ ^cells contained both p75^NTR+ ^and p75^NTR- ^cells, whereas those from p75^NTR- ^cells generated only p75^NTR- ^cells. In p75^NTR+ ^subpopulation, the percentage of p75^NTR+ ^cells declined time-dependently. In addition, p75^NTR+ ^cells could be serial passaged and generated p75^NTR+ ^β1-integrin^-^, p75^NTR- ^β1-integrin^-^and p75^NTR- ^β1-integrin^+ ^progenies (E), whereas p75^NTR- ^cells generated only p75^NTR- ^cells in 5th passage (F).

As shown in Figure 2 A~D, p75^NTR+ ^cells were detected in all 4 ESCC cell lines. The percentage of p75^NTR+ ^cells in Eca109, SHEC-1, SHEC-4 and SHEC-5 was 2.5%, 1.6%, 1.9% and 3.7%, respectively. To compare the growth characteristics of p75^NTR+ ^and p75^NTR- ^cells, equal numbers of sorted cells were cultured *in vitro *for up to 6 weeks. The percentage of p75^NTR+ ^cells decreased progressively during the first three weeks and maintained steady thereafter. The cells derived from p75^NTR+ ^cells contained both p75^NTR+ ^and p75^NTR- ^cells, whereas those from p75^NTR- ^cells generated only p75^NTR- ^cells.

In addition, the results of phenotypic analysis showed p75^NTR+ ^cells of different passages (total passages = 30) generated p75^NTR+^β1-integrin^-^, p75^NTR-^β1-integrin^- ^and p75^NTR-^β1-integrin^+ ^progenies (Figure 2E), whereas p75^NTR- ^cells generated only p75^NTR- ^cells even in the 30th passage (Figure 2F).

### Molecular characteristic of p75^NTR+ ^cells

**Figure 3:**
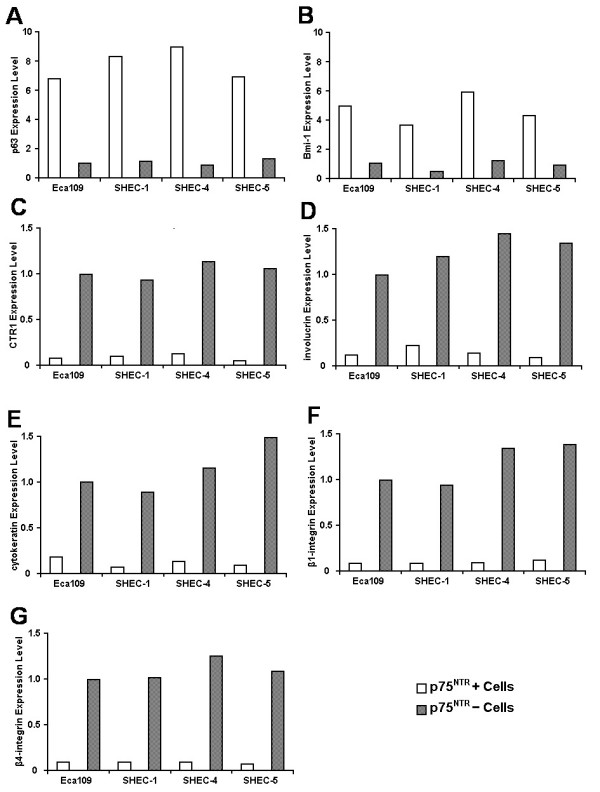
**Different gene mRNA expression in p75^NTR+ ^and p75^NTR- ^subpopulation**. mRNA expression level was measured by real-time qPCR. p63 (A) and Bmi-1 (B) were more intensely expressed in p75^NTR+ ^cells compared with p75^NTR- ^cells. Conversely, CTR1 (C), involucrin (D), cytokeratin 13 (E), β1-integrin (F) and β4-integrin (G) expression were lower.

As compared to p75^NTR- ^cells, p75^NTR+ ^cells were shown to differentially express the following genes (Figure 3 A~G): (a) p63, a marker identified in keratinocyte stem cells [22], was confined mainly to p75^NTR+ ^cells; (b) Bmi-1, a transcription repressor implicated in the regulation of self-renewal of normal and malignant stem cells [7,23], was significantly higher in p75^NTR+ ^cells; (c) copper transporter 1 gene (CTR1) [24], a specific transporter of copper associated with DDP resistance, was confined mainly to p75^NTR- ^cells. In addition, p75^NTR+ ^cells expressed lower levels of markers of differentiation such as involucrin, cytokeratin 13, β1-integrin and β4-integrin than p75^NTR- ^cells.

### Sphere-forming capacity of p75^NTR+ ^cells

**Figure 4:**
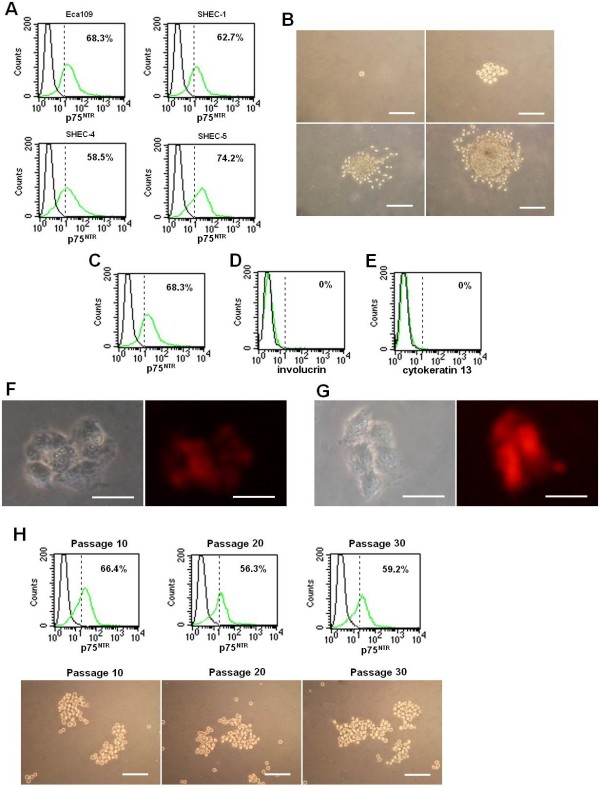
**Sphere formation in serum-free medium**. Tumor Cells formed nonadherent spheres in serum-free medium. The percentage of p75^NTR+ ^cells increased to 68.3% in Eca109, 62.7% in SHEC-1, 58.5% in SHEC-4 and 74.2% in SHEC-5 (A). Single p75^NTR+ ^cell could proliferate in serum-free culture and formed nonadherent cells sphere (B), contained both p75^NTR+ ^and p75^NTR- ^cells (C); p75^NTR+^cells obtained from the spheres did not expressed differentiated markers involucrin (D) and cytokeratin 13 (E). Floating p75^NTR+^cells could adhere and expressed involucrin (F) and cytokeratin 13 (G) in DMEM containing 10% fetal bovine serum. In addition, p75^NTR+ ^cells could be expanded as floating cell spheres for more than 30 passages, and the percentage of p75^NTR+ ^cells from passage 10, passage 20 and passage 30 was 66.4%, 56.3% and 59.2%, respectively (H). B and H bars = 100 μm. (F and G, bars = 50 μm)

Tumor cells were cultured in serum-free medium supplemented with growth factors. The formation of spheres were observed 10–15 days later. The percentage of p75^NTR+ ^cells was 68.3%, which was significantly higher than thatcultured in DMEM containg 10% FBS. Similar results were also obtained from SHEC-1 (62.7%), SHEC-4 (58.5%) and SHEC-5 (74.2%) (Figure 4A).

Sphere-forming capacity of p75^NTR+ ^cells isolated from Eca109 was examined. As shown in Figure 4B, single p75^NTR+ ^cell could proliferate in the serum-free medium. Three to 6 days after plating, the formation of spheres were observed. Two weeks later, the size of cell spheres became relatively large with the average diameter of 400 μm. Flow cytometry showed that the spheres contained both p75^NTR+ ^and p75^NTR- ^cells (Figure 4C). Meanwhile, p75^NTR+ ^cells obtained from spheres did not expresse mature markers involucrin (Figure 4D) and cytokeratin 13 (Figure 4E). Under differentiating condition (10% fetal bovine serum supplementation), the floating p75^NTR+ ^cells could adhere to the dish and form a single layer of confluent cells expressing involucrin (Figure 4F) and cytokeratin 13 (Figure 4G). Furthermore, p75^NTR+ ^cells from these spheres were able to passage repeatedly and the percentage of p75^NTR+ ^cells from passage 10, passage 20 and passage 30 was 66.4%, 56.3% and 59.2%, respectively (Figure 4H). In contrast, p75^NTR- ^cells were not able to form spheres.

### DDP sensitivity and ^64^copper accumulation assays

**Figure 5:**
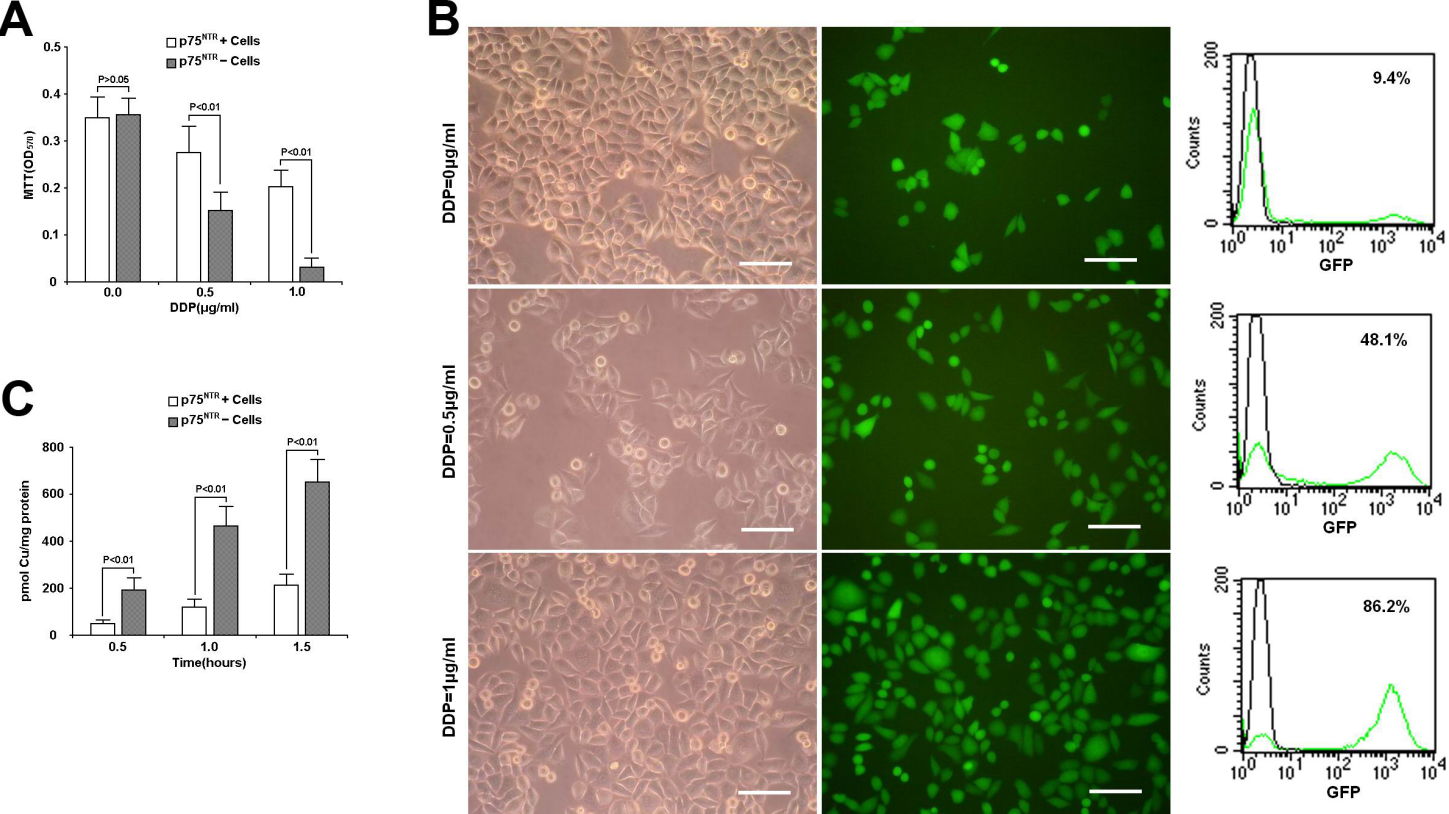
**DDP resistance of p75^NTR+ ^cells**. Treated with 0.5 and 1 μg/ml DDP for 4 days, the number of viable cells in p75^NTR+^subpopulation was significant higher than that in p75^NTR- ^subpopulation. (B) Enrichment assay showed that the fraction of eGFP-carrying cells derived from p75^NTR+ ^cells increased from 9.4% to 48.1% and 86.2%, respectively. (C) Copper accumulation was less in the p75^NTR+ ^cells than in the p75^NTR- ^cells at each time point. (B, bars = 100 μm)

Cell viability was measured by MTT assay 4 days after subjecting p75^NTR+ ^and p75^NTR- ^cells to DDP. The OD_570 _values of p75^NTR+ ^cells were 0.34 ± 0.06 (0 μg/ml, n = 3), 0.27 ± 0.08 (0.5 μg/ml, n = 3) and 0.19 ± 0.05 (1 μg/ml, n = 3), whereas the values of p75^NTR- ^cells were 0.36 ± 0.04 (0 μg/ml, n = 3), 0.16 ± 0.06 (0.5 μg/ml, n = 3) and 0.03 ± 0.03 (1 μg/ml, n = 3). These results suggested that the viability of p75^NTR- ^cells decreased significantly as compared to that of p75^NTR+ ^cells (P < 0.05, Figure 5A).

The DDP resistance of p75^NTR+ ^cells was further examined by detecting enrichment of eGFP labeled p75^NTR+ ^cells co-cultured with p75^NTR- ^cells following exposure to DDP. It was demonstrated that DDP induced significant enrichment of p75^NTR+ ^cells and their percentage increased from 9.4% to 48.1% and 86.2%, respectively (Figure 5B).

^64^copper accumulation assay of the p75^NTR+ ^and p75^NTR- ^cells was performed. The ^64^copper accumulation value of p75^NTR+ ^cells were 43.6 ± 12.0 pmol Cu/mg protein (0.5 h, n = 3), 120.7 ± 29.3 pmol Cu/mg protein (1 h, n = 3) and 208.6 ± 40.5 pmol Cu/mg protein (1.5 h, n = 3), whereas the values of p75^NTR- ^cells were 193.4 ± 49.1 (0.5 h, n = 3), 457.4 ± 87.7 (1 h, n = 3) and 658.2 ± 98.2 (1.5 h, n = 3). The result showed that ^64^copper accumulation in p75^NTR+ ^cells was significantly lower than that in p75^NTR- ^cells at each time point (P < 0.05 for all comparisons).

### Xenograft tumorigenicity assay

Various numbers of sorted Eca109 cells were suspended in 200 μl serum-free DMEM and injected subcutaneously into NOD/SCID mice. The results showed that at least 2 × 10^3 ^p75^NTR+ ^cells or p75^NTR- ^cells were needed to generate tumors. When transplanted cells were suspended in DMEM/Matrigel, the result showed that the lowest number of p75^NTR+ ^and p75^NTR- ^cells to generate tumors was 500 and 2 × 10^3^, respectively (Table 2).

**Table 2:** Tumorigenicity of p75^NTR+ ^ESCC in NOD/SCID mice xenograft

Cell numbers injected per mouse	DMEM		DMEM/Matrigel (1:1)	
	
	p75^NTR+^	p75^NTR-^	p75^NTR+^	p75^NTR-^
**100**	0/6	0/6	0/6	0/6
**500**	0/6	0/6	2/6	0/6
**2,000**	2/6	1/6	3/6	2/6
**10,000**	4/6	4/6	5/6	4/6
**100,000**	6/6	5/6	6/6	6/6

## Discussion

In the present study, we found that p75^NTR ^was confined to immature cells and absent in cells undergoing terminal differentiation in ESCC specimens. We also found the percentages of p75^NTR+ ^cells in the 4 ESCC cells lines ranged from 1.13% to 3.35%, which was similar to the percentages of CSCs as previously reported [1,25,26].

In agreement with the previous report [21], p75^NTR ^was confined to the basal layer in NEE. Although statistical analysis showed that positive rate of p75^NTR ^was not associated with lower pathological grade, histopathological analysis indicated that the number and distribution of p75^NTR+ ^cells altered depending on the degree of anaplasia in ESCC. In WDC, p75^NTR ^was confined to the surrounding basal-like cells which were at some distance from the centers of terminal differentiation. In MDC, the number of p75^NTR+ ^cells increased and their distribution became irregular with respect to the centers of terminal differentiation. In PDC, p75^NTR ^was diffusely distributed and there was virtually no relation between the distribution of p75^NTR+ ^cells and the small zones of terminal differentiation. Thus, both in NEE and ESCC, p75^NTR ^was mainly expressed in immature cells. Ki-67, a cell proliferation-associated nuclear antigen, was found in all stages of the cell cycle. Immunostaining for p75^NTR ^and ki-67 revealed that only a small proportion of p75^NTR+^cells expressed ki-67 in NEE, while the number of cancer cells coexpressing p75^NTR ^and ki-67 increased in ESCC. These results suggested that p75^NTR ^was expressed in cells that were proliferating or capable of proliferating. Furthermore, the distribution of p75^NTR ^in ESCC was similar to that of p63, a marker identified in keratinocyte stem cells [27,28]. Taken together, it could be deduced that p75^NTR ^was expressed in undifferentiated cells. Toinvestigate the identity of p75^NTR+ ^cells as CSCs in ESCC, we further studied the biological characteristics of p75^NTR+ ^cells in Eca109 and 3 newly established cell lines.

### Self-renewal capacity of p75^NTR+ ^cells

Self-renewal is one of the hallmarks of CSCs, which refers to the ability to form new stem cells with identical, and intact potential for proliferation, expansion, and differentiation. In the present study, our results revealed that p75^NTR+ ^cells could generate both p75^NTR+ ^and p75^NTR- ^progenies, but p75^NTR- ^cells could generate only p75^NTR- ^cells, suggesting a p75^NTR^-associated cell hierarchy may exist in ESCC.

Sphere-forming ability in serum-free medium was used to reflect the self-renewal capacity of CSCs in a variety of studies and sphere cells were widely regarded as CSCs [7,29-32]. In the present study, we found ESCC cells could form cell spheres and the percentage of p75^NTR+ ^cells increased significantly. p75^NTR+ ^cells could be propagated undifferentiatedly cells in serum-free medium and differentiate into involucrin^+ ^and cytokeratin 13^+ ^cells under differentiating conditions.

The higher expression of self-renewal associated gene and lower expression of differentiation markers also suggested that p75^NTR+ ^cells possessed the characteristics of ESCC CSCs.

### Chemotherapy resistance of p75^NTR+ ^cells

DDP is an important chemotherapeutic agent for human esophageal carcinoma though resistance to it is likely to develop during the therapy [33]. Reduced DDP accumulation is considered as the most important feature[34,35]. DDP enters cells much more slowly than most other classes of small-molecule anticancer agents. Current evidences showed that DDP uptake is mediated by CTR1, a major copper influx transporter in mammalian cells. Accumulated evidence indicates that CTR1 accountes for DDP uptake of cells in kinds of cancers including ovarian carcinoma, small-cell lung cancer, and prostate cancer.

The lower expression of CTR1 indicated p75^NTR+ ^cells might be more resistant to DDP than p75^NTR- ^cells. In addition, we used ^64^copper accumulation experiment to elucidate the DDP resistance because of the similar mechanism of DDP and copper uptake [36,37]. The lower ^64^copper accumulation also suggested that the low expression of CTR1 markedly impaired the influx of DDP in p75^NTR+ ^cells.

Meanwhile, the result of MTT assay showed that p75^NTR+ ^cells were more resistant to DDP than p75^NTR- ^cells in all 4 esophageal carcinoma cell lines. Furthermore, the result of the enrichment assay offered the direct evidence that p75^NTR+ ^cells were more resistant to DDP since p75^NTR+ ^and p75^NTR- ^cells were cocultured in the same condition.

### Xenograft tumorigenicity of p75^NTR+ ^

Finally, we injected 10^2 ^to 10^5 ^p75^NTR+ ^and p75^NTR-^cells isolated from Eca109 into NOD/SCID mice to determine whether p75^NTR+ ^cells possess higher tumorigenicity in immunocompromised mice than p75^NTR- ^cells. Although tumors were generated at similar cell doses (more than 2 × 10^3^) when p75^NTR+ ^cells or p75^NTR- ^cells were suspended in DMEM, higher tumorigenicity was shown by p75^NTR+ ^cells when the transplanted cells were suspended in DMEM/Matrigel before inoculation. The results confirmed the tumorigenicity of p75^NTR+ ^cells *in vivo*. Although p75^NTR- ^cells also exhibited some tumorigenic potential, this may be due to the meta-topical implantation of cells into a more permissive environment (e.g. subcutaneous site) compared to the environment of origin.

## Conclusion

In summary, we found that (1) p75^NTR ^was mainly confined to immature cells capable of proliferation and absent from cells which were undergoing terminal differentiation in ESCC. (2) p75^NTR+ ^cells isolated from ESCC cell lines were able to generate both p75^NTR+ ^and p75^NTR- ^cells, to form spheres in serum-free medium supplemented with growth factors, and to differentiate into mature esophageal squamous epithelial cells. and (3) p75^NTR+ ^cells were more resistant to DDP than p75^NTR- ^cells. Our results demonstrated that p75^NTR+ ^cells possessed some characteristics of CSCs, namely, self-renewal and chemotherapy resistance.

## Abbreviations

CSCs: cancer stem cells; ESCC: esophageal squamous cell carcinomas; KI: ki-67 index; FACS: fluorescence-activated cell sorting; CTR1: copper transporter 1; IC10: the 10% inhibitory concentration; IC50: the 50% inhibitory concentration; IC90: the 90% inhibitory concentration; NOD/SCID mice: congenitally immune-deficient nonobese diabetic/severe combined immune-deficiency mice; NEE: normal esophageal epithelial; WDC: well-differentiated cases; MDC: moderately differentiated cases; PDC: poorly differentiated cases.

## Competing interests

The authors declare that they have no competing interests.

## Authors' contributions

ZYX conceived the design of the study and was in charge of its coordination. SDH established ESCC cell lines, participated in data analysis, performed data interpretation and drafted the manuscript. YY carried out the genetic analysis, participated in animal experiments and helped to draft the manuscript. XHL participated in histopathological studies, performed the statistical analysis and helped to draft the manuscript. DJG participated in cell culture. CGB participated in immunohistochemical studies and the data analysis. FW participated in flow cytometry analysis and fluorescence-activated cell sorting. JHL carried out the drug sensitivity assay. All authors read and approved the final manuscript.

## Pre-publication history

The pre-publication history for this paper can be accessed here:

http://www.biomedcentral.com/1471-2407/9/9/prepub
